# Intensive West Nile Virus Circulation in Serbia in 2018—Results of Integrated Surveillance Program

**DOI:** 10.3390/pathogens10101294

**Published:** 2021-10-08

**Authors:** Tamaš Petrović, Milanko Šekler, Dušan Petrić, Dejan Vidanović, Zoran Debeljak, Gospava Lazić, Diana Lupulović, Mihaela Kavran, Milena Samojlović, Aleksandra Ignjatović Ćupina, Bojana Tešović, Sava Lazić, Mišo Kolarević, Tatjana Labus, Boban Djurić

**Affiliations:** 1 Department for Virology, Scientific Veterinary Institute “Novi Sad”, 21000 Novi Sad, Serbia; goga@niv.ns.ac.rs (G.L.); diana@niv.ns.ac.rs (D.L.); milena.s@niv.ns.ac.rs (M.S.); lazic@niv.ns.ac.rs (S.L.); 2 Specialized Veterinary Institute “Kraljevo”, 36000 Kraljevo, Serbia; sekler@vsikv.com (M.Š.); vidanovic@vsikv.com (D.V.); debeljak@vsikv.com (Z.D.); tesovic@vsikv.com (B.T.); kolarevic@vsikv.com (M.K.); 3 Laboratory for Medical and Veterinary Entomology, Faculty of Agriculture, University of Novi Sad, 21000 Novi Sad, Serbia; dusan.petric@polj.uns.ac.rs (D.P.); mihaela.kavran@polj.uns.ac.rs (M.K.); cupinas@polj.uns.ac.rs (A.I.Ć.); 4 Veterinary Directorate, Ministry of Agriculture, Forestry and Water Management, 11000 Belgrade, Serbia; tatjana.labus@minpolj.gov.rs (T.L.); boban.djuric@minpolj.gov.rs (B.D.)

**Keywords:** West Nile virus, mosquito surveillance, wild bird surveillance, horse surveillance, WNV national surveillance program 2018

## Abstract

The results of the Serbian national integrated West Nile virus (WNV) surveillance program conducted in 2018 and funded by the Serbian Veterinary Directorate are presented. The WNV surveillance program encompassed the entire territory of Serbia and was conducted by the veterinary service in collaboration with entomologists and ornithologists. The objective of the program was early detection of WNV circulation in the environment and timely reporting to the public health service and local authorities to increase clinical and mosquito control preparedness. The program was based on the detection of WNV presence in wild birds (natural hosts) and mosquitoes (virus vectors) and on serological testing of sentinel horses (WNV-specific IgM antibodies). The season 2018 was confirmed to be the season of the most intensive WNV circulation with the highest number and severity of human cases in Serbia ever reported. The most intense WNV circulation was observed in the northern and central parts of Serbia including Vojvodina Province, the Belgrade City area, and surrounding districts, where most positive samples were detected among sentinel animals, mosquitoes and wild birds. The majority of human cases were preceded by the detection of WNV circulation during the surveillance. The WNV surveillance program in 2018 showed satisfactory results in the capacity to indicate the spatial distribution of the risk for humans and sensitivity to early detection of WNV circulation in the environment.

## 1. Introduction

The West Nile virus (WNV) is a zoonotic neurovirulent mosquito-borne Flavivirus, which is maintained in nature in an enzootic transmission cycle between avian hosts and ornithophilic mosquito vectors. Besides birds as natural hosts, the virus occasionally infects other vertebrates including humans and horses, which are incidental, dead-end hosts. Unfortunately, in humans and horses, WNV may cause sporadic disease outbreaks that may result in fatal outcomes. WNV is nowadays considered one of the most widespread flaviviruses in the world and is endemic in Africa, Asia, Europe, the Middle East, Australia, and the Americas [1,2]. 

Until the beginning of the 1990s, WNV was essentially maintained in endemic cycles in Africa, India, the Middle East and Europe and only affected humans and horses sporadically with rare reports of encephalitis [3,4]. In Europe, WNV epidemiological behaviour changed when it re-emerged in Romania in 1996, Russia in 1999 and the Mediterranean area, causing dozens of human and equine deaths [1,5,6,7]. In addition, from 2004, strains belonging to WNV lineage 2 have been identified in Europe [8,9,10,11], and since 2008, WNV has been heavily spreading throughout central and south-eastern Europe, representing a serious veterinary and public health problem [12,13]. 

Before 2009, the WNV status in Serbia was mostly unknown and the virus presence remained unrecognized. The first confirmation of the presence of WNV circulation was obtained through the findings of 12% (42/349) WNV neutralizing antibody-positive horses sampled during 2009–2010 in the northern part of the country. WNV antibody-positive horses were found in 14 out of 28 municipalities studied, and spread over 200 km [14]. In the coming years, more studies were conducted on WNV serology in horses and each year increasing seroprevalence (up to 49.29%) as evidence of more intensive virus circulation was observed [15,16]. The first detection of the presence of WNV in mosquito vectors in Serbia was observed in 2010 in the city of Novi Sad when WNV lineage 2 strains were detected in 3 out of 841 pools of mosquitoes collected from 66 localities in 29 settlements in Vojvodina Province during the period 2005–2010 [17]. In addition, the first detection of WNV (lineage 2 strains) in mainly resident wild birds was observed in Serbia in early spring 2012, when WNV antibodies were detected in 7.6% (7/92) of blood sera samples, and the presence of the virus was confirmed in 8 out of 81 (9.87%) tissue samples of 133 found dead and live captured wild birds in Vojvodina Province [18].

The history of WNV infection among the human population in Serbia is mostly unknown and/or unrecognized, probably due to the low disease prevalence before 2012. The first serological studies conducted among the human population in Serbia revealed a low prevalence for WNV antibodies, being ≤ 8% from the early 1970s to 1990 [19,20]. After a gap of many years, the prevalence of 3.99% (18/451) in samples of human sera collected from 2005 to 2010 in Vojvodina Province was established, indicating possibly low or sporadic circulation of WNV in Serbia in the past [17]. In 2012, the first outbreak of WNV infection in clinical and epidemic form in humans was reported in Serbia [21,22]. A total of 42 West Nile fever cases were clinically and laboratory-confirmed out of 69 reported ones, and 9 cases resulted in fatal outcomes. All cases were detected in the central and northern parts of the country [22,23]. From 2012, human outbreaks and cases of West Nile fever have been reported each year, at a lower or higher extent. The WNV epidemic was more severe during 2013, with 302 reported and 202 laboratory-confirmed human cases, and 35 lethal outcomes [24]. In 2018, the biggest West Nile fever epidemic ever reported in Serbia with a high number of human cases was observed. In total, 415 human cases were reported from 17 out of 25 districts of Serbia, and 36 lethal outcomes [25,26].

Except for the continuous surveillance of human cases in the frame of the human medicine data service that was established from 2012, official integrated program-based WNV surveillance did not exist in Serbia before 2014. Based on the obtained results and the anticipated intense circulation of WNV, which poses substantial risks for both public and animal health in Serbia, the Veterinary Directorate of the Ministry of Agriculture, Forestry and Water Management launched and funded a national integrated WNV surveillance program starting from 2014. The results of the surveillance program conducted during 2014 and 2015 showed the success of the program in the early detection of WNV in the environment, as well as some difficulties and shortcomings that have already been published [27]. Each year, the program is updated according to the newly available data (surveillance results from previous years and results obtained in research studies) and funding possibilities. In this article, we present the methodology of the implementation and the obtained results of the national integrated WNV surveillance program conducted during 2018, as the year with the most intensive WNV circulation in Serbia until now. 

## 2. Results

**Figure 1:**
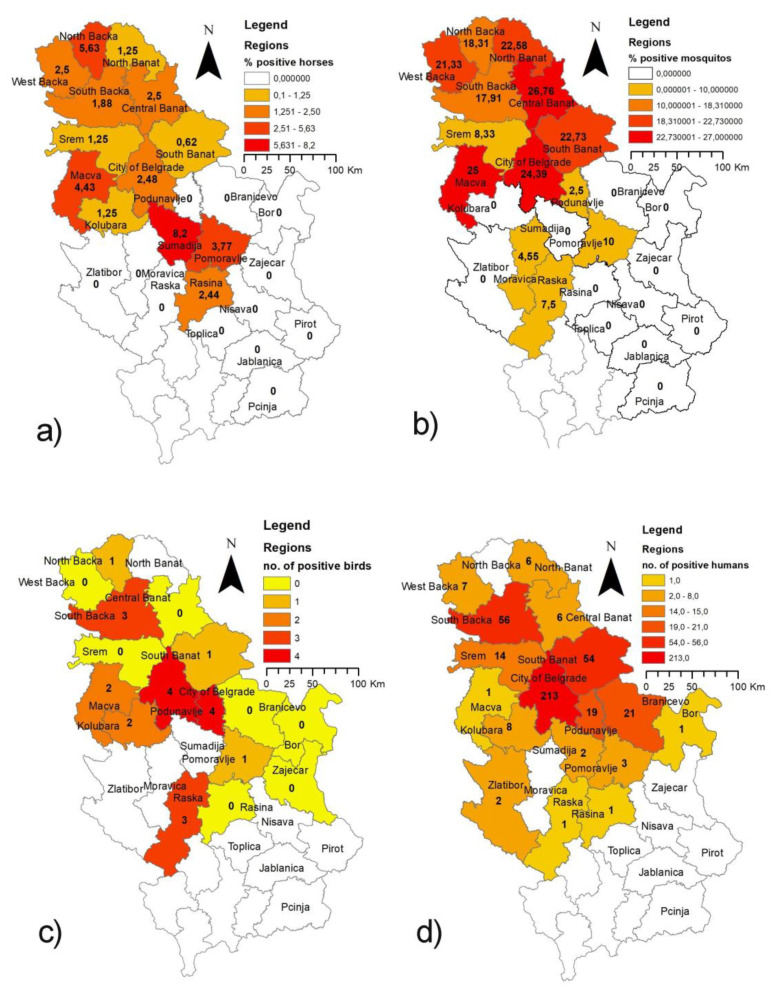
Distribution of WNV activity detected during the 2018 surveillance program at the district level. (**a**,**b**) show WNV infection rates as percentages (number of positive samples/number of samples tested) for a horses, and b mosquito pools. The comma instead of the point in the percentage values represent the decimal separator shown by the software that was used for the preparation of the maps. (**c**) presents the number of found WNV positive wild birds and (**d**) presents the number of clinical and laboratory-confirmed human West Nile cases reported to the ECDC. The basic administrative maps were extracted from the GADM database of Global Administrative Areas (www.gadm.org), version 3.6 (accessed on 15 February 2021), and were changed subsequently according to the data presented in them.

**Figure 2:**
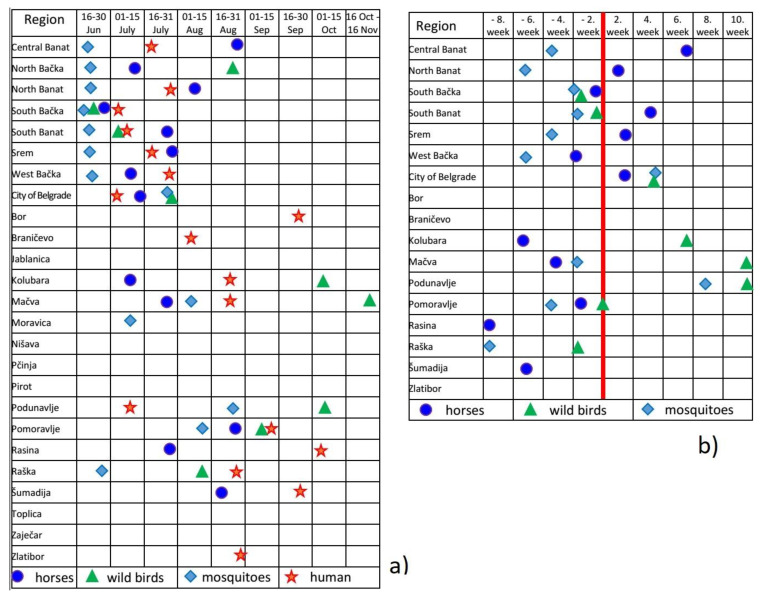
The temporal (time-line) distribution of the first positive results detected during WNV surveillance and the time of the first West Nile human cases in 2018. (**a**) The first positive results obtained during WNV surveillance and the first reported cases of human West Nile infection in 2018 are represented by symbols per district level. The top seven districts represent the Vojvodina Province, followed by the other districts of central Serbia are sorted in alphabetical order; (**b**) the time of the first positive results in horses, mosquitoes, and birds obtained during surveillance in 2018 concerning the time of the first human cases is shown. The red line represents the time when the first human case was detected per district level, and positive findings are presented in 2-week intervals periods in columns before and after the first human case was detected. Only the districts where human cases were detected are presented.

Detailed results of the WNV surveillance program in Serbia during 2018 and a comparison of the surveillance data of all three tested pillars (horses, wild birds, and vector mosquitoes) obtained during the WNV surveillance program with the human WNV-positive cases reported to the ECDC in 2018 [26] are presented in Table 1 and Figure 1 and Figure 2.

Between June and September 2018 in Serbia, positive serological responses, i.e., the presence of WNV IgM antibodies in horse blood sera, were detected in 1.75% (44/2511) of tested animals. Positive serological responses in horses were detected in all four testing months (June–September) with the highest prevalence in the period of most intense vector mosquitos’ activity. The first WNV IgM antibody-positive horse was detected on 30th June. The percentage of WNV IgM antibody-positive animals ranged per month of testing from 0.79% (5/630) in September to 2.78% (18/648) in July. The number of IgM antibody-positive horses and the number of districts in which the positive horses were detected per each month of testing are presented in Table 2. Detected percentages of positively reacted/tested horses per district and the date of the first positive serological response per district are presented in Table 1 and Figure 1 and Figure 2. In passive surveillance, clinical signs of neurological dysfunction in one horse were reported in Belgrade in July 2018 [28].

During the surveillance of WNV presence in the environment, 846 pools of *Cx. pipiens* mosquitoes were tested from June to August 2018 (during 4 sampling periods at 2-week intervals), and WNV was confirmed in 107 (12.65%) samples. The first WNV positive mosquito pool samples were detected on 19th June. The prevalence of WNV in mosquitoes increased after the first positive findings in June (11.21%; 24/214), while 15.05% (59/392) mosquito samples tested positive in July, and 10% (24/240) in August. Positive mosquito samples were detected in 13 out of the 25 districts (52%). The prevalence of WNV in mosquitoes and the number of positive districts per each month of testing are presented in Table 2. In positive districts, the percent of detected WNV positive samples ranged between 2.50% (Podunavlje) to 26.76% (Central Banat). In a total of 6 (24%) districts, more than 20% of examined mosquito samples tested positive for WNV (Figure 1). The percentage of WNV-positive/tested mosquito pool samples per district and the date of the first WNV-positive mosquito pool sample detected per district are presented in Table 1 and Figure 1 and Figure 2.

In total, 301 samples of wild birds were tested. Out of this number, tracheal/pharyngeal swabs of 198 live-trapped wild birds, tissue samples from 79 found dead, and tissue samples from 24 shot wild birds were tested. The first WNV positive wild bird (common kestrel (*Falco tinnunculus*)) was detected on 20th June in the South Bačka District. Among 79 samples of found dead wild birds, WNV was detected in 8 (10.13%) cases, and among 198 tested tracheal/pharyngeal swabs of 198 live-trapped wild birds, WNV was detected in 6.56% (13/198) samples. The presence of WNV was detected in tissues or tracheal/pharyngeal swab samples of carrion crows (*Corvus corone*), hooded crows (*Corvus cornix*), Eurasian magpies (*Pica pica*), common kestrels (*Falco tinnunculus*), Eurasian jay (*Garrulus glandarius*), common blackbird (*Turdus merula*), long-eared owl (*Asio otus*) and great tit (*Parus major*). All of them are resident birds in Serbia. The presence of WNV was not detected in any of 24 samples of shot carrion crows and Eurasian magpies (12 in July and 12 in September) in the West Bačka District. In total, WNV was detected in 6.98% (21/301) of tested wild birds. WNV positive wild birds were detected during each month of testing and were found in 9 out of the 25 districts of Serbia (Table 2), mostly in the northern and central parts of the country. The number of WNV-positive/tested wild bird samples detected per district and the date of the first WNV-positive wild bird sample detected per district are presented in Table 1 and Figure 1 and Figure 2.

In line with the main goal of the integrated WNV surveillance program, that is, early detection of WNV circulation in the area, the presence of WNV in Serbia was detected as early as 19th June in mosquitoes, which was at the same time the first round of mosquito sampling in 2018. At that time, WNV presence was detected in mosquitoes from 4 districts, and after 2 or 3 days in the mosquitoes from additional 3 districts, out of 25 districts in Serbia. All of these 7 WNV positive districts represent the Vojvodina Province, the northern part of the country. By the end of June, in the rest of Serbia, only one more district (Raška) tested positive for the presence of WNV in mosquitoes. The first detection of WNV in wild birds was in the sample of tracheal/pharyngeal swab of the ill common kestrel (*Falco tinnunculus*) on 20th June, and the first IgM antibody positive horse was detected on 30th June, both in the South Bačka District (Vojvodina Province). These results preceded the onset of the first human cases of West Nile disease (WND) in Serbia (detected on July 2nd in South Bačka and South Banat Districts) by 2 weeks (mosquitoes and wild bird’s data) or 2 days (IgM antibody positive horse). Positive findings in the frame of integrated WNV surveillance program preceded human infection at district level by detection of WNV presence in one, two or in all three tested pillars (horses, mosquitoes or wild birds) in 12 out of 17 or 15 districts where human WNV infection was suspected or confirmed consequently. On a district level, the first positive data on WNV presence obtained in the surveillance program were detected from 2 months to a few days before the first human WND case. Contrary to that, the integrated WNV surveillance program was not successful in early warning before the first WNV infection in humans in 5 districts, which was either due to the delayed detection of WNV presence in horses, mosquitoes or wild birds (in two Districts) or to the absence of positive findings on WNV presence during the surveillance activity (in three Districts). The detailed data obtained per each district were presented in Figure 2 and Table 1.

## 3. Discussion

The design and the obtained results of the integrated WNV national surveillance program funded by the Veterinary Directorate of the Ministry of Agriculture, Forestry, and Water Management conducted in 2018 in Serbia are presented. The surveillance program was successful in detecting WNV presence and circulation about 2 weeks (from 2 months to a few days) before the onset of the first reported/confirmed human case in most (12/17 or 12/15) of the districts where human WND cases were detected. This suggests the success of the program in 2018. However, some shortcomings of the surveillance program were observed, which will be discussed below.

The main objective of the program was the early detection of WNV circulation in the country, followed by timely alerting of public health services and local authorities to increase both clinical and mosquito control preparedness. For those purposes, we developed the surveillance program considering different strategies previously used for WNV surveillance [13,29,30,31,32,33,34]. Similarly to what was done during 2014 and 2015 [27], the integrated national WNV surveillance program conducted in 2018 in Serbia was based on the testing and data collection from three pillars: sentinel animals–horses (detection of the presence of WNV IgM antibodies as a result of recent WNV infection), mosquitoes as vectors and wild birds as virus natural hosts that were tested for the WNV presence. 

**Figure 3:**
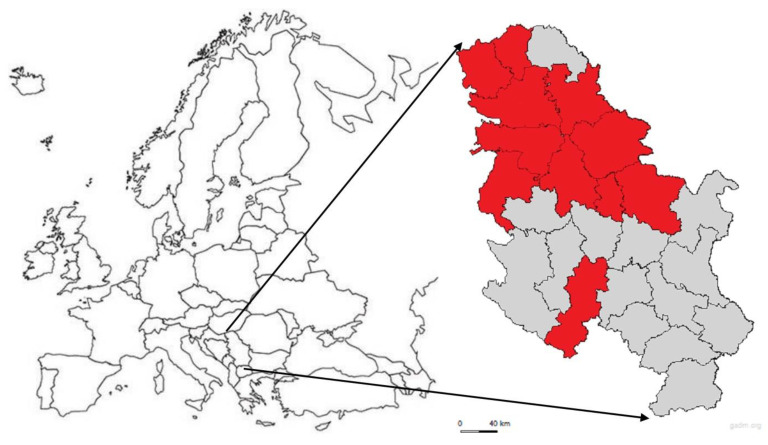
Categorization of districts in the Republic of Serbia according to the risk of WNV outbreak. The figure shows the geographic position of the Republic of Serbia in Europe. The districts (NUTS3) with a higher risk of WNV infection are marked in red (11 districts), and the districts with a lower risk of WNV infection are unmarked (grey). The solid black lines in the maps of the Republic of Serbia represent the borders of districts (all 25 districts in Serbia that were under WNV surveillance). The most northern seven districts represent the Autonomous Province of Vojvodina. Description of the sampling locations: districts names, geographic coordinates and web links are provided as the supporting information (Table S1). The basic administrative maps were extracted from the GADM database of Global Administrative Areas (www.gadm.org), version 3.6 (accessed on 15 February 2021), and later on, changed according to the data presented in them.

Although highly susceptible, horses are less suitable for use as sentinel animals in areas where vaccination of the horse population against WNV is widely used. In Serbia, horses are not vaccinated against WNV, and therefore, they were included as sentinel animals in WNV surveillance like it was done in other WNV surveillance programs [32,34]. Horses with an unknown history of previous WNV infection that were included in WNV surveillance were tested on the presence of WNV IgM antibodies following the results of earlier studies on WNV seroprevalence in horses in Serbia [14,15,16,27], categorization of districts in Serbia as of high or lower risk of WNV infection (Figure 3) and the surveillance plan (Table 3). Besides the sentinel animals, direct monitoring of WNV presence was done by testing wild birds as natural hosts of WNV and mosquitoes as virus vectors. Wild birds examined during the surveillance included species that are highly susceptible to WNV infection in various environmental conditions throughout the world as well as in the Serbian region [8,18,33,35] and have been efficiently used in WNV surveillance systems [27,33,36,37]. For the monitoring of WNV presence in vectors, only the *Cx. pipiens* (including biotypes pipiens, molestus and their hybrids) was tested since the results of all previous studies suggested that this species is the most competent WNV vector in Serbia as well as in the surrounding regions [6,17,38,39]. The WNV surveillance program based on these three surveillance pillars (horses, wild birds and mosquitoes) was conducted during the period from June to August (mosquitoes), to September (horses) or to October (wild birds), as the period of most intensive vector activity during the year [13,39], except for the found dead wild birds in high-risk districts, where the testing of wild birds on WNV presence has been done from June until the end of the year (Table 3).

The WNV surveillance program revealed the highly intense circulation of WNV in Serbia during 2018, the same as it was confirmed in the whole region [40,41,42,43,44,45]. The first evidence of WNV circulation was obtained by detection of the virus in mosquitoes on 19th June, simultaneously in 4 districts, and 2 to 3 days later in an additional 3 districts. At the same time, it was the first round of mosquito collection and testing within the WNV surveillance program. Such results pointed out that the first collection and testing of mosquitoes probably has not been done on time, suggesting that an earlier start of mosquito sampling and testing should have been planned, probably in May. A limited funding possibility in 2018 resulted in missing such an early sampling period (May), which significantly influenced the capacity for early detection of WNV in the environment. Nevertheless, even with the late start, the program provided quite good results in the early detection of WNV circulation. Approximately at the same time as the first mosquito pools tested positive for WNV, the first WNV positive tracheal swab of a wild bird was detected (on 20th June). A few days later, the first WNV IgM antibody-positive horse (30th June) was identified. All these positive findings of WNV circulation were located in all 7 districts of Vojvodina Province, a northern part of Serbia, which has previously been identified as a region with intensive WNV circulation in the past years [14,16,17,18,27]. However, unlike previously implemented surveillance programs in 2014 and 2015 [27], as well as during 2017, besides 7 Serbian northern districts of Vojvodina Province and Belgrade district, i.e. the usual areas of intensive WNV circulation, the results from 2018 revealed more intensive WNV circulation also in the districts of central, eastern and western parts of Serbia. In total, WNV circulation was detected in 16 out of 25 Serbian districts. Positive findings were recorded in all three surveillance pillars in 6, in two surveillance pillars in 7 and in only one surveillance pillar in 3 districts (Figure 1 and Figure 2, and Table 1 and Table 2).

The extraordinary intensive circulation of WNV was also confirmed through the number/percentage of positive findings. The obtained results showed that the presence of WNV IgM antibodies in horse blood sera was detected in 1.75% (44/2511) of tested animals in 13 (52%) out of the 25 districts in Serbia and during all four months of surveillance (Table 2). Virus presence was detected in 12.65% of tested mosquito samples in 52% (13/25) Serbian districts and the WNV prevalence in mosquito samples of more than 20% was detected in 6 out of 25 districts in Serbia. This situation could be explained by high temperatures in April and May 2018, with the highest ever recorded average April temperatures recorded in Serbia since 1888 [40]. A similar situation was observed by the testing of wild birds. WNV was detected in 10.13% (8/79) tissue samples of found dead wild birds and in 6.56% (13/198) of tested pharyngeal swabs of live wild birds. WNV positive wild birds were detected during each month of testing from June until November and were found in 9 out of 25 districts of Serbia, overlapping with the districts with WNV positive findings in mosquitoes and horses. All detected WNV positive wild birds belonged to the resident bird species in Serbia, confirming the conclusions from previous studies [18,27] that WNV is endemic in Serbia.

It should be emphasized that 2018 was also the year of the highest ever reported number and severity of human WNND cases in Serbia [25,26,45]. In total, 415 registered human WND cases, out of which 365 laboratory-confirmed cases, were reported. The lethal outcome was confirmed in 36 cases. Almost all cases were detected in the central and northern parts of the country, and 337 out of 415 (81.2%) of them were detected in just four districts (Belgrade, South Banat, South Bačka and Srem) [25,26]. The highest number of human cases was reported in the Belgrade region (213; 51.33%); then, in South Bačka (56, 13.49%), South Banat (54; 13.01%) and Srem districts (14; 3.37%) of Vojvodina Province, in the northern part of Serbia. Additionally, high numbers of human cases were registered in districts Podunavlje (19; 4.58%) and Braničevo (21; 5.06%) belonging to the central and central-eastern part of Serbia. In total, human WNV cases were reported in 17 (68%) out of 25 districts in Serbia. In 2 districts (districts Rasina and Raška) where per one human clinical case was reported, they were not laboratory-confirmed (Figure 1, Table 1) [25,26]. The 2018-season in Serbia was characterized by an unusually early start and increased transmission of WNV, same as in the entire Balkans region and whole Europe [25,26,40,41,42,43,44,45]. The first human case was reported on the 2nd of July and the last one by the middle of November 2018. As early as July, about 70 human cases were registered in 8 districts (8/25; 32%) [25].

The majority of human cases were preceded by the detection of WNV in horses, wild birds, and mosquitoes (Figure 2, Table 1). Surveillance reports of WNV activity/presence in horses, wild birds and mosquitoes preceded human cases in their district of residence from a couple of days to 8 weeks in 12 (70.59%) out of 17 districts, from which the human cases were reported. Out of that number, human WNV infection was preceded by WNV positive findings in mosquitoes from 2 to 8 weeks in 9 districts, by WNV positive wild birds detected from one day to 2 weeks in 3 districts, and by WNV IgM antibody positive horses detected from 2 days to 8 weeks before the first human case in 7 districts. In 5 (29.42%) districts, the surveillance program was not successful in view of early detection of WNV circulation before the first human WNV infection, and in 3 of them, no positive results during the surveillance have even been detected (Figure 2, Table 1). The possible reasons for the failure of early WNV detection in those districts could be connected with the late start of the surveillance program activities. One of those districts was the Belgrade city area with more than 200 reported human cases in 2018 and a known history of intensive WNV circulation for many years back. In that area, the first human case was reported 2 weeks before the WNV positive results in horses, and almost one month before WNV positive results in mosquitoes and wild birds. As already mentioned, the WNV surveillance program in 2018 started in June, but considering a very early WNV circulation in the environment in the 2018 season, the start of the surveillance activities needed to be shifted to May. Such a situation also indicates the need to include meteorological data and predictions [46] into the risk assessment during the preparation and implementation of the WNV surveillance program for each year. Interestingly, in another district—Braničevo District, with a high number of reported human WNV cases (21)—all findings obtained during the integrated WNV surveillance were negative. This could be partly explained by the fact that due to the proximity of Belgrade, many residents of Braničevo District work in the city, often staying for the whole day in the city. It is possible that WNV infection of those human cases originated from Belgrade. However, this situation also points to the need to check and, if needed, change the sampling locations in this and some other districts according to the residence locations of the human cases. In 2 other districts, Bor and Zlatibor, where the WNV surveillance findings were also negative, only 1 and 2 human cases were reported, respectively. Since the data of their travel history were not available, it cannot be stated with certainty that those 3 persons were infected in their district of residence. The other possibility, i.e., that the surveillance program was not sensitive enough to detect the lower intensity of WNV circulation, is less probable, considering that positive indications of WNV circulation were obtained in 2 more districts (North Bačka and Moravica), where no human cases were reported. In one of them (North Bačka), WNV positive findings were detected in all three surveillance pillars (horses, wild birds and mosquitoes) (Figure 2, Table 1).

The districts of Vojvodina Province and the City of Belgrade have previously been identified as areas with high WNV circulation [14,15,16,17,18,27]. Geographical and climatic features of that area could be a possible explanation for that. The entire territory of Vojvodina Province and Belgrade City is located in the flatland area of up to 250 m altitude characterized by a hot summer continental climate (*Dfa* climates) with an average temperature in the warmest month of ≥22 °C, as well as by the presence of many slow water flows (Danube, Tisza, and Sava rivers), swamp areas and small lakes that are the favourite habitats of many resident wild bird species, but also for shorter or longer stays of many migratory birds on their migration routes. It is highly probable that in the past, migratory birds have introduced WNV into the aforementioned areas, which also represent suitable breeding sites producing abundant populations of *Cx. pipiens,* the most competent virus vector [17,39]. Consequently, this ornithophilic mosquito species became infected and further transmitted the virus to the resident birds as natural hosts [18]. Nowadays, WNV infection could be considered endemic in northern and central parts of Serbia, and it will likely continue to pose a significant problem and challenge for animal and human health. In addition, the first detection of the Usutu virus as a new zoonotic flavivirus in mosquitoes in Serbia [43,47] additionally complicated the situation and highlighted the necessity for redesigning the existing WNV surveillance program.

Satisfactory results obtained in 2018 are the consequence of the implementation of an integrated WNV surveillance program designed, validated and improved in close collaboration between veterinary service, ornithologists and entomologists based on the “One Health” strategy. Although the "One Health" principle is essential in the management of vector-borne and zoonotic diseases [45,48,49], and the integrated use of data for early warning and inter-sectoral priority setting is pioneeristic, the early warning system before human case occurrence is recurrently not operationally prioritized in many countries [45,50]. The results of the presented program were continuously communicated by the Veterinary Directorate to the Serbian CDC (National Institute of Public Health), which further disseminated the surveillance results and information on citizen personal protection measures to the regional institutes of public health and district’s public health authorities, as well as to the municipality authorities. In that way, the overall population has been informed about the risk of contracting the disease and the need to protect themselves from insect bites with repellents or mosquito nets. Vector- and nuisance mosquito control is under the jurisdiction of the municipalities, and although some steps were taken to harmonize and centralize the mosquito control in Serbia, still there is no national strategy for responding to early warnings about the risk of vector-borne disease outbreaks.

Although the WNV surveillance program in 2018 proved very successful, we need to point out two major drawbacks. Previous years of virus circulation in the area were characterized by a high percentage of already seropositive horses, so the adequacy of the number of seronegative (sentinel) horses that can be used in the WNV surveillance program in the following years is questionable. This problem can be overcome by the inclusion of some other animal species as sentinel animals for indirect monitoring of WNV presence in the program, such as cattle and pigs that are mentioned in the literature as potential sentinel animals [12,51]. For this reason, intensive testing of young cattle and swine (born after the previous mosquito season) for this purpose was done during 2018 and 2019 with promising results, but these results will be presented in another article. The second drawback of the program was connected to the non-uniformity in the collecting of wild bird samples (both the pharyngeal swabs of trapped live birds and tissues samples from found dead wild birds). Some samples were collected in June, but the majority of samples were collected from September to November, with a relatively small number of samples in July and August. This is mainly attributed to the summer holidays season and the low active manpower at that time of the year, as well as to the lower activity of ornithologists in that time of the year due to the wild birds’ biology. Testing of wild birds at the end of the vector activity season, when WNV activity, as well as the most of human cases has already been present/detected in most of the regions, has no practical value. A possible improvement in this part of the WNV surveillance program could be reached through more intensive surveillance of chosen highly susceptible wild bird species with ornithologists and hunters, in predefined localities during the period May–August.

Collaboration between veterinary and human health services as well as medical entomologists and ornithologists, and the coordination and management of data at both national and local levels, are the prerequisites for a successful program and essential in providing early warning and adequate protection of human and animal health. Based on the obtained results and anticipated continued intense circulation of WNV, further studies, especially those on the introduction of other animals species (e.g., cattle and pigs) as sentinel animals, usage of meteorological data [46] and studies on local temperature and humidity predictions as well as continuous monitoring and surveillance of WNV infection and virus epidemiology in Serbia in the coming years, will be of vital importance toward further enhancement of early detection capacity and sensitivity, and improvement of the capacity to indicate the spatial distribution of the risk for WNV circulation and human and animal infection.

## 4. Materials and Methods

### 4.1. WNV Surveillance Program

The WNV national integrated surveillance program, established by the Veterinary Directorate of the Serbian Ministry of Agriculture, Forestry and Water Management, designed as both active and passive surveillance, has been conducted starting from the year 2014 [4,25]. In the preparation of the yearly WNV surveillance programs, the existing knowledge about WNV transmission and epidemiology was incorporated, and the parameters of different program models and guidance [12,13,29,30,31,32,33,52,53,54] were studied. In addition, each year before the vector season, modifications regarding new data from previous seasons and new perspectives were incorporated as an update of the program for the ongoing season. The main objectives of the program were early detection of WNV circulation in nature and the consequent provision of timely information to veterinary services and human epidemiologists to establish protective measures for human and animal health and guide mosquito control efforts. The WNV surveillance program in 2018 was based on serology testing of horses that were used as sentinel animals, whereas wild birds and mosquitoes were tested on the presence of the virus by molecular methods, as a part of an active surveillance program. Passive surveillance encompassed serological and virological examinations of clinically ill horses manifesting signs of neurological dysfunction. The program encompassed the entire territory of Serbia and was conducted by veterinary institutes and field veterinary services in close collaboration with entomologists and ornithologists. The selection and distribution of sampling sites for active surveillance in each district were defined according to the assessment of the risk of exposure to WNV. According to the surveillance results from the previous years and previous WNV outbreaks data, the territory of Serbia was divided into districts with a higher or lower risk of WNV presence (25 districts–NUTS3 level, Figure 3). The number of tested samples was defined at the level of each district to the risk of WNV infection. 

### 4.2. Serological Surveillance of Sentinel Horses

During the 2018 season, the serology surveillance of horses was performed by detection of WNV IgM antibodies. The horses were sampled as described in Table 2 and tested on the presence of WNV IgM antibodies by commercial ELISA tests (*INgezim West Nile IgM* from Ingenasa and *ID Screen West Nile IgM Capture* from IDVet) following the manufacturer’s instructions.

### 4.3. WNV Surveillance in Wild Birds and Mosquitoes

Dead wild birds found in the natural environment, particularly the resident species most susceptible to infection, e.g., from the order Passeriformes, mainly *Corvidae* (magpie, crow, raven, rook., etc.), raptors (northern goshawk, falcon, and eagle), common pheasants and songbirds, as well as birds that died in rehabilitation centres, zoos or bird breeding farms (mostly raptors such as falcons, eagles, and hawks) being submitted for testing for the presence of WNV. The selected and collected wild bird species were previously confirmed as the bird species in which WNV has often been detected in previous years in this part of Europe [8,18,55]. The collection of tissue samples of found dead wild birds in the environment, shot targeted wild birds (corvids; mainly Eurasian magpies), and tracheal/pharyngeal swabs of live-trapped wild birds (during the ringing and other activities of bird protection societies), was conducted as described in Table 2.

Mosquitoes were sampled over 4 sampling periods, at approximately 2-week intervals from June to the end of August, by dry-ice baited traps without light (trap types NS2), operating overnight (on average, 14 hours, from afternoon to the next day morning) in semi-urban and urban localities, as described in Table 2. Mosquitoes were collected, put on dry ice in the field and maintained under cold conditions throughout the testing process. They were identified to the species level, counted and pooled according to the date, location and species. *Culex pipiens* mosquitoes (including biotypes pipiens, molestus and their hybrids), previously indicated as the primary WNV vector species in this region [13,17], were tested in pools of up to 50 mosquitoes. One mosquito pool was tested per sampling site for each date of collection. In cases when the number of *Cx. pipiens* mosquitoes in the traps exceeded 300 individuals, 2 mosquito pool samples were tested per sampling site.

Tissues from wild birds found dead and wild birds that were shot (corvids; mainly Eurasian magpies) during surveillance and tracheal/pharyngeal swabs of live-trapped wild birds, as well as mosquito pools, were tested for the presence of WNV RNA as described in Table 2.

### 4.4. WNV Genome Detection by Molecular Methods

The preparation of samples for molecular detection of WNV genomic RNA was performed as follows: Tissue samples (brain, spleen, lung) of wild birds in amounts of 0.2 g or up to 50 individual mosquitoes (one pool) were placed in 2 mL microtubes and homogenized in 1 mL sterile phosphate-buffered saline for 5 min using a TissueLyser LT (Qiagen, Hilden, Germany) operating at 50 Hz. The homogenates were then centrifuged for 10 min at 2000× *g*, and the supernatant was used for RNA extraction. Tracheal swabs from wild birds were immersed in 0.75 mL of sterile phosphate-buffered saline during the sampling, placed on ice packs during the transport to the lab, and after a reception in the lab, vortexed vigorously for 3 minutes. The supernatants were directly used for RNA extraction. 

Mosquito pools and bird samples (tissues and tracheal swabs) were tested for WNV RNA presence by TaqMan-based one-step reverse transcription real-time PCR (RT-qPCR) that amplified both lineage 1 and 2 strains. Viral RNA was extracted using the commercial kits (*QIAamp cador Pathogen Mini Kit* (Qiagen, Germany) and *IndiSpin Pathogen Kit* (Indical Bioscience GmbH, Germany) according to the manufacturer’s instruction. One-step RT-qPCR was conducted using the commercial kits *RNA UltraSense™ One-Step qRT-PCR System* or *SuperScript™ III Platinum™ One-Step qRT-PCR Kit* (Invitrogen^TM^, ThermoFisher Scientific) with the primers and probes that targeted the nucleocapsid protein C gene regions of WNV, as described by Linke et al. [56] and NS2A gene region described by Eiden et al. [57].

### 4.5. Human Cases

Data from records of human cases used for the evaluation of horse, bird, and mosquito WNV surveillance represent the number of clinical and laboratory-confirmed human West Nile disease (WND) positive cases reported by the Serbian CDC (Institute of Public Health of Serbia–Batut) [25] to the European Centre for Disease Prevention and Control (ECDC) in 2018 [26].

## Figures and Tables

**Table 1:** Cumulative results of WNV surveillance program in Serbia during 2018, and comparison Table 2018.

Region–District (NUTS3 Units)	Horses-Blood Sera forAnti-WNV IgM Ab			Mosquitoes(*Culex pipiens*)			Wild Birds (Tissues and Tracheal Swabs)			Human Cases *	
	Tested	Positive	First Positive Reported	Tested Pools	Positive Pools	First Pos. Pool Reported	Tested	Positive	First Positive Reported	First Case Reported	Total No Cases
Central Banat	160	4	30/08	71	19	**19/06**	3	0	/	16/07	6 (5)
North Bačka	160	9	13/07	71	13	21/06	5	1	28/08	/	-
North Banat	80	1	07/08	31	7	**22/06**	0	0	/	30/07	6 (6)
South Bačka	160	3	**30/06**	67	12	**19/06**	21	3	**20/06**	02/07	56 (50)
South Banat	161	1	27/07	66	15	**19/06**	54	1	**01/07**	02/07	54 (50)
Srem	160	2	31/07	48	4	**19/06**	4	0	/	16/07	14 (14)
West Bačka	160	4	**13/07**	75	16	**21/06**	25	0	/	30/07	7 (6)
City of Belgrade	161	4	15/07 **	41	10	31/07	52	4	31/07	**02/07**	213 (197)
Bor	80	0	/	20	0	/	3	0	/	**25/09**	1 (1)
Braničevo	115	0	/	40	0	/	29	0	/	**06/08**	21 (11)
Jablanica	80	0	/	20	0	/	0	0	/	/	-
Kolubara	80	1	**11/07**	/	/	/	20	2	04/10	21/08	8 (2)
Mačva	158	7	**30/07**	16	4	**06/08**	30	2	16/11	21/08	1 (1)
Moravica	51	0	/	22	1	11/07	0	0	/	/	-
Nišava	80	0	/	20	0	/	0	0	/	/	-
Pčinja	80	0	/	20	0	/	0	0	/	/	-
Pirot	80	0	/	20	0	/	0	0	/	/	-
Podunavlje	63	0	/	40	1	27/08	27	4	06/10	**09/07**	19 (17)
Pomoravlje	53	2	**30/08**	20	2	**14/08**	3	1	11/09	11/09	3 (3)
Rasina	41	1	**31/07**	20	0	/	1	0	/	02/10	1 (0)
Raška	87	0	/	40	3	**29/06**	20	3	**13/08**	28/08	1 (0)
Šumadija	61	5	**18/08**	20	0	/	0	0	/	25/09	2 (1)
Toplica	80	0	/	20	0	/	0	0	/	/	-
Zaječar	80	0	/	20	0	/	4	0	/	/	-
Zlatibor	40	0	/	18	0	/	0	0	/	**28/08**	2 (1)
**25**	**2511**	**44**		**846**	**107**		**301**	**21**			**415 (365)**
***%***		**1.75**			**12.65**			**6.98**			

* Human cases reported by Serbian CDC (Institute of Public Health of Serbia–Batut) to the ECDC in 2018: number of human cases (laboratory-confirmed cases) available at: http://www.batut.org.rs/index.php?content=1742 (accessed on 7 October 2021) and https://www.ecdc.europa.eu/en/publications-data/transmission-west-nile-virus-june-december-2018-table-cases-2018-transmission(accessed on 7 October 2021); ** Acute neuroinvasive clinical case in the horse. 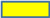
—positive results obtained during surveillance that preceded human infections at the districts (NUTS3) level. 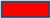
—human infections at the district (NUTS3) level where no previous positive findings were obtained during surveillance.

**Table 2:** The temporal distribution of the obtained results of the WNV surveillance program in Serbia during 2018.

	June	July	August	September	October	November/December	Total
Horses tested	587	648	646	630	nd	nd	2511
Horses WNV IgM Ab positive	7 (1.19%)	18 (2.78%)	14 (2.17%)	5 (0.76%)	nd	nd	44 (1.75%)
Districts with WNV IgM positive horses	4 (16%)	8 (32%)	7 (28%)	3 (12%)	nd	nd	13 (52%)
Mosquito pools tested	214	392	240	nd	nd	nd	846
Mosquito pools WNV positive	24 (11.21%)	59 (15.05%)	24 (10%)	nd	nd	nd	107 (12.65%)
Districts with WNV positive mosquito pools	8 (32%)	11 (44%)	8 (32%)	nd	nd	nd	13 (52%)
Wild birds tested	13	27	81	53	57	70	301
Wild birds WNV positive	2 (15.39%)	2 (7.41%)	3 (3.7%)	4 (7.55%)	5 (8.77%)	5 (7.14%)	21 (6.98%)
Districts with WNV positive wild birds	1 (4%)	2 (8%)	3 (12%)	3 (12%)	3 (12%)	2 (8%)	9 (36%)

nd: not done.

**Table 3:** WNV surveillance plan (sampling and testing) in 2018.

	Lower-Risk Districts											Higher-Risk Districts												
**Testing of sentinel horses for WNV IgM antibodies (ELISA)**																								
Sentinel horses	‑ up to 20 horses ‑ minimum of 5 localities per district ‑ testing on four occasions (once per month in June, July, August, and September)											‑ up to 40 horses ‑ minimum of 8 localities per district ‑ testing on four occasions (once per month in June, July, August, and September)												
**RT-qPCR detection of WNV in natural reservoirs and vectors**																								
Wild birds	‑ up to 20 dead wild birds found from May through October											‑ up to 30 found dead throughout the year, or ‑ up to 30 samples of purposely shot or live-trapped susceptible bird species during the period May–October												
Mosquitoes (*Culex pipiens*)	‑ collection at 2–3 week intervals ‑ 5 localities per district ‑ 4 samplings from June to August (2 samplings in July)											‑ collection at 2–3 week intervals ‑ 10 localities per district ‑ 4 samplings from June to August (2 samplings in July)												
**Sampling strategy**																								
Sampling distribution	Jan	Feb	Mar	Apr	May	Jun	Jul	Aug	Sep	Oct	Nov	Dec	Jan	Feb	Mar	Apr	May	Jun	Jul	Aug	Sep	Oct	Nov	Dec
Horses																								
Mosquitoes																								
Wild birds																								

The different colours only representing/highlighting horses, mosquitoes or wild birds.

## Data Availability

Publically available data on human WND cases reported by Serbian CDC (Institute of Public Health of Serbia–Batut) to the ECDC in 2018 can be found here: http://www.batut.org.rs/index.php?content=1742 (accessed on 7 October 2021) and https://www.ecdc.europa.eu/en/publications-data/transmission-west-nile-virus-june-december-2018-table-cases-2018-transmission (accessed on 7 October 2021). The data obtained within the frame of WNV surveillance program can be provided by the corresponding author (tomy@niv.ns.ac.rs) upon reasonable request.

## Supplementary Materials

The following are available online at https://www.mdpi.com/article/10.3390/pathogens10101294/s1, Table S1: Description of the sampling locations: Districts names, geographic coordinates, and web links.
